# Co-Development of a Bereavement Support Program for Parents With Lived Experience of Stillbirth or Neonatal Death in Pakistan

**DOI:** 10.1192/bjo.2024.122

**Published:** 2024-08-01

**Authors:** Subia Naz, Tazeen Ali, Nasim Chaudhry, Nusrat Hussain, Tracey Mills

**Affiliations:** 1 Aga Khan University, Karachi, Pakistan; 2 Pakistan Institute of Living and Learning, Karachi, Pakistan; 3 University of Manchester, Manchester, United Kingdom; 4 Liverpool School of Tropical Medicine, Liverpool, United Kingdom

## Abstract

**Aims:**

Rates of stillbirth and neonatal deaths are high in low- and middle-income countries including Pakistan and these are one of the most stressful life-events for parents and families. Society does not appropriately recognize perinatal loss and support from healthcare professionals is often very limited or non-existent in Pakistan. Therefore, we aimed to co-develop and assess the feasibility of a bereavement support program for parents who experienced stillbirth/neonatal death in a public health facility in Pakistan.

**Methods:**

This study adopted a sequential mixed-method design. The first phase involved co-development of a bereavement support program through a consensus process involving multidisciplinary health professionals, stakeholders and parents with previous experience of perinatal death (n = 23) using the Nominal Group Technique. Phase 2 includes a feasibility assessment using before and after cohort design. Sixty women (30 per phase) with recent experience of stillbirth and/or neonatal death will be recruited, from a public hospital in Pakistan. The main outcome measures will include recruitment and retention and acceptability of the study processes and data collection.

**Results:**

Following the consensus process, agreed intervention components included an educational workshop for healthcare staff, creation of a bereavement champion group of health workers in the facility and offering post-natal telephone peer support to bereaved mothers. The educational component for healthcare staff includes Advance Bereavement Care (ABC) workshop for all staff and bereavement champions (n = 15 healthcare workers) who later received one day's training and one-day training refresher. This component aims to improve care, act individually and as a group to identify areas for development, encourage good practice and support colleagues. The peer support component includes telephone support provided by women with previous experience of perinatal death (n = 7) trained by the research team. Supervision arrangements are in place for champions and peer supporters. The feasibility study is ongoing.

**Conclusion:**

The co-development process ensured the cultural relevance of both components of the bereavement support program. The process also contributed to improving the sense of ownership by healthcare facility. Feasibility study will confirm whether parents are willing to take part, acceptability and whether future research to assess the effectiveness of the intervention on improving care after SB/NND is feasible.

